# Height-obesity relationship in school children in Sub-Saharan Africa: results of a cross-sectional study in Cameroon

**DOI:** 10.1186/s13104-015-1073-4

**Published:** 2015-03-26

**Authors:** Lifoter K Navti, Uta Ferrari, Emmanuel Tange, Klaus G Parhofer, Susanne Bechtold-Dalla Pozza

**Affiliations:** CIHLMU Center for International Health at Ludwig-Maximilians-Universitaet, Munich, Germany; Department of Biochemistry, Catholic University of Cameroon (CATUC), P.O. Box 782, Bamenda, Cameroon; Diabetes Research Group, Department of Medicine IV, Ludwig-Maximilians Universitaet, Ziemssenstr. 1, 80336 Munich, Germany; Department of Food Science and Technology, Catholic University of Cameroon (CATUC), P.O. Box 782 Bamenda, Cameroon; Department of Medicine II - Grosshadern, Ludwig-Maximilians Universitaet, Marchioninistr. 15, 81377 Munich, Germany; Pediatric Endocrinology and Diabetology, University Children’s Hospital, Ludwig-Maximilians Universitaet, Lindwurmstr. 4, 80337 Munich, Germany

**Keywords:** Height, Waist circumference, BMI

## Abstract

**Background:**

In developed nations, taller children exhibit a greater propensity to overweight/obesity. This study investigates whether this height-adiposity relationship holds true for Cameroon children using two parameters of adiposity including body mass index (BMI) and waist circumference (WC).

**Methods:**

In 557 children (287 boys and 270 girls, mean age 9.0 ± 1.8 years) from the North West Region of Cameroon height, weight and WC were measured and BMI calculated. Variables were converted to standard deviation scores (SDS). Participants were divided into quartiles of height SDS, then mean of age and sex-standardized body fat parameters compared across quartiles. The frequency of excess adiposity was calculated within each quartile. Correlation and regression analysis were used to assess height-adiposity relationships.

**Results:**

Multiple comparisons indicated a significant increase in mean BMI (−0.08 to 0.65) and WC (−0.11 to 0.87) SDSs with increasing quartiles of height SDS. Frequency of overweight/obesity and abdominal overweight/obesity was highest among children with highest height SDS (30.2 – 33.1%) and lowest in their shortest peers (0.7 – 5.0%). There was a linear relationship between height SDS and BMI SDS (R^2^ = 0.087, *p* < 0.001); height SDS and WC SDS (R^2^ = 0.356, *p* < 0.001) among both boys and girls.

**Conclusions:**

This study shows that in Cameroon just as in developed economies a higher height SDS is associated with a higher frequency of overweight/obesity. This is independent of the parameter used to evaluate overweight/obesity (BMI SDS or WC SDS).

## Background

There is a global increase in the prevalence of overweight and obesity [1] now affecting approximately 10% of school children worldwide [2,3] This increase had been noted to occur at a faster rate in developing countries undergoing nutrition transition [4,5]. Available data on preschool children indicate that the prevalence of overweight in 1991 was 2.9% for Cameroon [6]. However, newer data especially on school-age children are not available for Cameroon. This neglect could be due to the fact that it is still a belief that overweight and obesity are problems of the developed economies. This raises concerns as it is well known that obesity is associated with an increased risk of hypertension [7], cardiovascular disease [8], type 2 diabetes [9] and respiratory diseases [6]. Several studies have suggested that the propensity to an irregular growth pattern could explain, to some degree, overweight and obesity in children [10-12].

It is therefore interesting to look at the interaction of height and weight status. Thus, a recent study in Chile, a transition economy, indicated that there is a positive association between obesity and higher height-for-age in children [5]. Also, between 1988 and 2003, serial cross-sectional surveys among 3 year old English children showed that the increase in obesity was greatest among the tallest children [13]. In addition, similar analyses have shown tallness to be associated to thicker skinfolds in children [14] and that height (during childhood) in obese children is independently associated with adult body mass index (BMI) [15]. Thus, the authors concluded that childhood height monitoring could be useful in identifying children at risk of becoming overweight or obese.

There are a number of different measures of adiposity, which can be used clinically and epidemiologically. All have advantages and shortcomings. BMI is the widely accepted measure to determine weight status in both children and adults. Until recently, many studies had relied on the National Center for Health Statistics/World Health Organisation (NCHS/WHO) international growth reference, which had its own practical problems. The WHO has now established new BMI reference curves for children, which includes data from six countries [16]. Even though BMI is quick to measure and non invasive [17], there is documented evidence of its limitations. BMI does not differentiate between fat mass and fat-free mass [18], it’s unable to indicate fat distribution in the body [19] and it has been shown to have a low sensitivity; thus it can misclassify a high proportion of children with excess adiposity [20]. Despite these limitations, a population-based study indicated that the prediction error from other measures of body fatness (skinfold thicknesses or bioelectrical impedance) were comparable to those obtained when BMI was used to establish age and sex prediction formulae for percentage body fat (% BF) in children and adults [21].

Waist circumference is a measure of abdominal fat and provides information about fat distribution. It can be used as an index of adiposity; it is easy to determine with high reproducibility [22]. Further, excess abdominal fat has been associated to adverse obesity related outcomes [23]. However, the lack of a universally accepted site of measurement makes international comparison of this measure difficult [24]. In addition, waist circumference has gained little attention as an important measure of adiposity in Sub-Saharan African countries.

The aim of this project was to evaluate whether the association between increased height and obesity observed in developed countries can also be seen in children in Cameroon and whether the relationship holds true for waist circumference.

## Methods

### Subjects

This study was a cross sectional analysis of data collected from children of ages 5 to 12 years from randomly selected schools (public and private) in both rural and urban settings of the North West Region of Cameroon. A total of 557 school-age children (270 girls and 287 boys) formed the study population. A list of both public and private primary schools was obtained from the North West Regional Delegation for Basic Education and 6 schools were chosen at random and contacted by the principal investigator (L.K.N). In cases when the school administration refused to participate, another school was chosen at random within the area. A quota sampling procedure was used in each school.

### Anthropometric measurements

All measurements were carried out on school premises (on appointment dates fixed by the school authorities) by the principal investigator (L.K.N) and trained nurses. Before each child was measured consent was obtained from the school head teacher and parents. Assent was also obtained from each child before measurement. All measurements were taken between 7 and 9 am.

Height was measured to the nearest 0.1 cm using a portable stadiometer (Seca 213, Germany). Body weight was measured using a digital scale (Omron BF 511, Japan) to the nearest 0.1 kg. These measurements were taken with children putting on light school uniforms without shoes. Body Mass Index (BMI) was calculated as weight (kg) divided by height squared (m^2^) [17].

Waist circumference (WC) was measured in the middle of the 10th rib and the iliac crest to the nearest millimetre with subjects wearing only underwear by a single trained measurer. Readings were recorded with the subjects at a standing position and at the end of a normal expiration [24] using a non-elastic flexible body circumference measuring tape (Seca 201, Germany).

Undernutrition was assessed using the WHO classification system [25]. The intra-class (within school) correlation for anthropometric variables in this study was 0.550.

### Ethical issues

Ethical approval was obtained from the Institutional Review Board of the Biotechnology Center of the University of Yaoundé I. Also, administrative clearances were obtained from the Public Health and Basic Education Regional Delegations of the North West Region of Cameroon. Parents gave written informed consent before any study related procedure was performed.

### Statistical analysis

Statistical procedures were performed using SPSS for Windows version 16.0 and data was checked for normality using the Kolmogorov-Smirnov (K-S) test. Metric variables were reported as mean (95% CI). Standard deviation scores (SDS) for height, weight and BMI were calculated using the WHO AnthroPlus software, which makes use of the WHO 2007 reference data for children [16]. Also, the SDSs for WC were calculated by making use of the UK reference data for WC [24]. The unadjusted and age-adjusted means of anthropometric variables were compared between boys and girls using a *t*-test for independent groups. The WHO cut-off points were used to determine the prevalence of overweight (> + 1SD) and obesity (> + 2SD). In addition, the 91st percentile of WC [24] was the cut-off point to determine the prevalence of excess abdominal fat.

The study participants were then divided into quartiles of height SDS. A 1-way ANOVA with post-hoc Bonferroni test was used to compare mean BMI and WC SDSs and other variables between quartile groups and the prevalence estimates within each quartile of height SDS calculated. Pearson correlation coefficients were used to assess the relation between height and the two parameters of adiposity. This relationship was further demonstrated using the most appropriate linear regression models containing type of school (private and public), area of residence (rural and urban setting), age and height SDS to predict/quantify the difference in body fatness between boys and girls at different levels of height SDS using the two adiposity parameters. In these models, age, school and area of residence had no significant association. Also, non-linearity was assessed using residual plots. The plots obtained did not show any increment or diminution in the spread of residuals. Different interaction terms were included in these models to assess for effect modification.

A *p*-value of 0.05 was considered to be statistically significant.

## Results

Table 1 shows the descriptive characteristics of the study population according to gender, with almost equal representation of boys and girls. There was no statistical significant difference in height and weight between boys and girls when these variables were unadjusted and age-adjusted. On an absolute scale, there was also no significant difference in BMI by gender. However, on an age adjusted basis, boys had a significantly higher BMI SDS while girls had a significantly higher WC SDS.

**Table 1:** **Characteristics of study population according to gender [mean (95% CI)]**

**Variables**	**Girls**		**Boys**		***p*** **-value**
	**N = 270**		**N = 287**		
Age	8.9	(8.7 - 9.1)	9.0	(8.8 - 9.2)	0.400
Height (cm)	132.4	(131.0 - 134.1)	133.0	(131.6 - 134.4)	0.536
Height SDS^a^	0.01	(−0.16 - 0.18)	−0.07	(−0.22 - 0.08)	0.494
Weight (kg)	30.8	(29.8 - 31.8)	30.8	(29.9 - 31.7)	0.998
Weight SDS^a^	0.13	(−0.01 - 0.27)	0.19	(0.07 - 0.31)	0.518
BMI (kg/m^2^)	17.2	(16.9 - 17.5)	17.1	(16.9 - 17.3)	0.478
BMI SDS^a^	0.19	(0.07 - 0.31)	0.38	(0.29 - 0.47)	0.015
WC (cm)	58.4	(57.7 - 59.1)	58.6	(58.0 - 59.2)	0.679
WC SDS^b^	0.51	(0.40 - 0.62)	0.36	(0.27 - 0.45)	0.039

^a,^ ^b,^standard deviation scores (SDS) account for sex and age and differences represent values in this study relative to the WHO 2007 and UK 1990 growth reference data respectively. Abbreviations: BMI, body mass index; WC, waist circumference.

**Figure 1:**
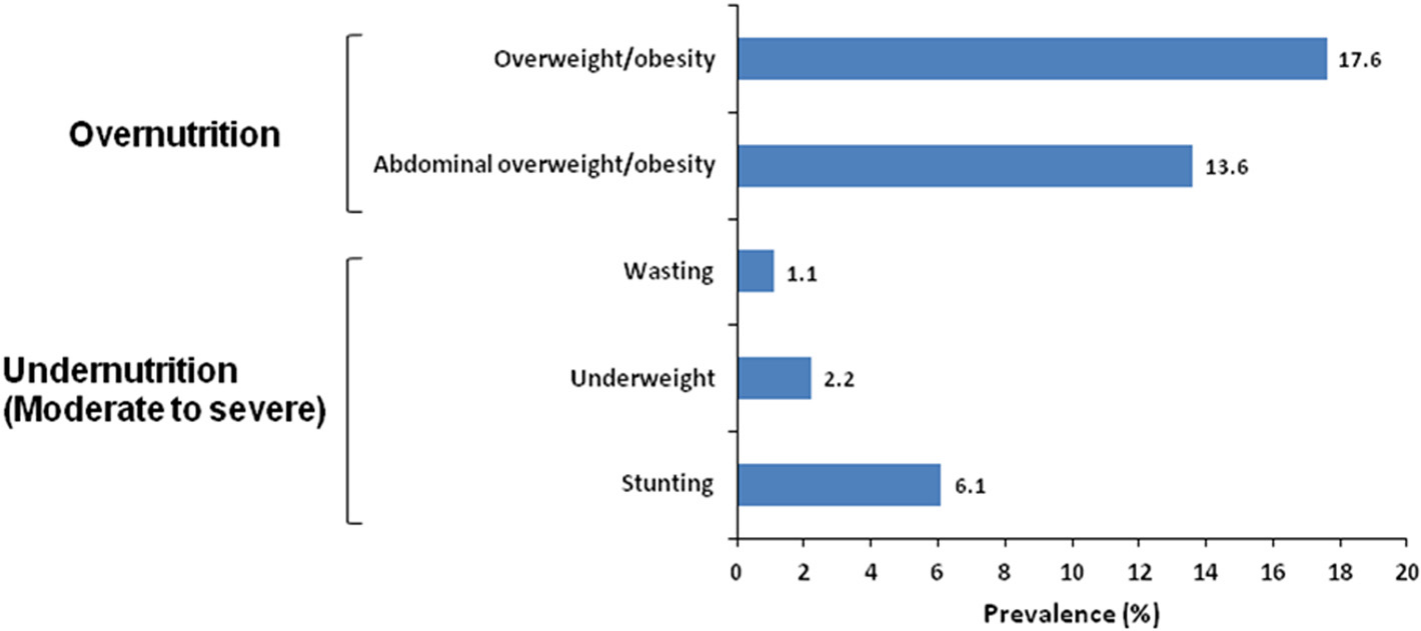
**Nutrition status of the study population showing the distribution of overnutrition and undernutrition (< −2 SDS of weight-for-height, weight-for-age and height-for-age for wasting, underweight and stunting respectively).**

The distribution of overweight and obesity within the investigated population was 14.7% and 2.9% respectively when BMI SDS was used to categorise, while the distribution was 13.6% on the basis of WC SDS (Figure 1).

When the children were divided according to height quartiles (mean heights: Q1: 125.6 cm, Q2: 131.1 cm, Q3: 135.1 cm, Q4: 139.1 cm), mean BMI and BMI-SDS (Table 2) as well as mean WC and WC-SDS (Table 3) increased significantly (*p* < 0.05) with increasing height. Similarly, prevalence of overweight/obesity increased in girls and boys (Tables 2 and 3).

**Table 2:** **Mean BMI SDS and prevalence of overweight/obesity across quartiles of height SDS**

**Quartiles of height SDS**	**N**	**Mean height (cm) (95%** **CI)**	**Mean height SDS (95%** **CI)**	**Mean BMI SDS (95%** **CI)**	**Prevalence of overweight/obesity**		
					**Overall % (95%** **CI)**	**Girls % (95%** **CI)**	**Boys % (95%** **CI)**
1	140	125.6 (124.1-127.1)	−1.71* (−1.82- -1.60)	−0.08* (−0.14-0.02)	5.0 (2.4-9.9)	4.3 (1.5-11.3)	5.7 (2.3-13.8)
2	141	131.1 (129.4-132.8)	−0.43* (−0.47- -0.39)	0.29* (0.14-0.44)	19.1 (13.5-26.4)	16.2 (9.2-26.7)	21.9 (13.9-32.7)
3	137	135.1 (133.3-136.9)	0.36* (0.32-0.40)	0.35* (0.22-0.48)	20.4 (14.6-27.9)	25.0 (16.2-36.4)	15.9 (9.1-26.4)
4	139	139.1 (137.1-1.41.1)	1.70* (1.55-1.85)	0.65* (0.48-0.82)	33.1 (25.8-41.3)	31.3 (21.3-43.4)	34.7 (24.9-45.9)

CI, confidence interval; SDS, standard deviation score. Prevalence was estimated using the WHO 2007 BMI cut-off.

*p < 0.05 after One-way ANOVA with post-hoc Bonferroni test.

**Table 3:** **Mean WC SDS and prevalence of abdominal overweight/obesity across quartiles of height SDS**

**Quartiles of height SDS**	**N**	**Mean WC (cm) (95%** **CI)**	**Mean WC SDS (95%** **CI)**	**Prevalence of abdominal overweight/obesity**		
				**Overall % (95%** **CI)**	**Girls % (95%** **CI)**	**Boys % (95%** **CI)**
1	140	56.5 (55.9 - 57.1)	−0.11* (−0.22 - 0.00)	0.7 (0.1 - 3.9)	0.0 (0.0 - 1.0)	1.4 (0.2 - 7.6)
2	141	58.8 (57.9 - 59.7)	0.45* (0.31 - 0.59)	11.3 (7.8 - 16.3)	16.2 (9.2 - 26.7)	6.8 (2.5 - 15.5)
3	137	58.9 (58.1 - 59.8)	0.52* (0.40 - 0.64)	12.4 (7.6 - 19.3)	17.6 (10.4 - 28.2)	7.2 (3.0 - 16.9)
4	139	60.0 (58.9 - 61.0)	0.87* (0.72 - 1.02)	30.2 (23.0 - 38.5)	39.1 (28.1-51.3)	22.7 (14.4 - 33.6)

CI, confidence interval; WC, waist circumference; SDS, standard deviation score. Prevalence was estimated using the 91st centile WC cut-off.

*p < 0.05 after One-way ANOVA with post-hoc Bonferroni test.

**Figure 2:**
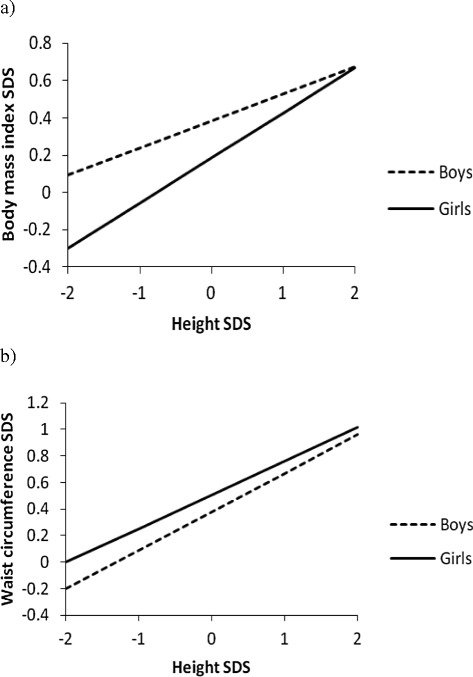
**Relationship between obesity parameters and height. a)** Relation between BMI and height. Boys (R^2^ = 0.061, *p* < 0.001); Girls (R^2^ = 0.115, *p* < 0.001). **b)** Relation between WC and height. Boys (R^2^ = 0.231, *p* < 0.001); Girls (R^2^ = 0.154, *p* < 0.001).

In our study population, the correlation coefficients (*r*) between height and the two parameters of obesity ranged from 0.246 to 0.481. This association was significant at the 0.05 level for boys and girls regardless of the obesity parameter used. Further demonstration of this association was carried out by using linear regression models containing height SDS and age as independent variables to predict levels of the measures of adiposity in both boys and girls. In all two measures of adiposity, the associations were significant (*p* < 0.001). Figure 2a shows the association between height SDS and BMI SDS for both boys (R^2^ = 0.061) and girls (R^2^ = 0.115), confirming that the BMI was highest among the tallest children. At all levels of height SDS, BMI was higher for boys than girls.

Equivalent findings (as seen in Figure 2b) were observed when height SDS was used to predict levels of WC SDS for boys (R^2^ = 0.231) and girls (R^2^ = 0.154), confirming that the tallest children accumulated more abdominal fat that their shortest peers. However, the girls had a higher waist circumference than boys at all levels of height SDS. That notwithstanding, this gender difference was not as pronounced as that for BMI.

For all the two measures of adiposity, the mean residual was 0 for both boys and girls. Also the standard deviation of the mean residual for boys ranged from 0.80 to 0.81 and that for girls was from 0.83 to 0.94. This indicates a distribution that is almost or near normal.

## Discussion

This current study confirms that with increasing quartiles of height SDS, mean BMI SDS and WC SDS increased, which corresponded to a significant increase in the prevalence of overweight/obesity and central overweight/obesity. In other words, this indicates that regardless of the parameter of obesity, the highest prevalence is in the top quartile of height SDS. The prevalence of overweight/obesity in the fourth quartile of height SDS was more than six times that of the first quartile. Also, this study confirms a positive association between height and obesity assessed by BMI and WC in both boys and girls.

The contribution of height to overweight/obesity is gaining more recognition and there is increasing evidence suggesting that taller children tend to be overweight/obese [5,13,14]. It was formerly thought this association was typical when BMI is used to assess body fatness. Like in some developed nations, this study has shown that equivalent relationships are also obtained when WC is used, suggesting that tallness is likely a predictor of excess adiposity. However, larger studies (including data from other regions of Cameroon) are still needed to confirm these findings.

There are different explanations for this height-adiposity relationship. From a dietary point of view, early high protein intake has been hypothesized to account for the fact that children of today tend to be taller and heavier. For instance, a French study on 10-year old children showed that mean energy intake dropped between 1978 and 1995 as a result of a decrease in fat and carbohydrate intake. However, over the same period percentage energy from protein increased, children had a higher stature, accumulated more abdominal fat and there was an 8.1% increase in obesity among children [26]. There is no data available to assess if similar nutritional changes could explain our observations in our sample of school age children from the North West Region of Cameroon. Another study has demonstrated that a high protein intake during the early years of life (<24 months) is associated with increased obesity (assessed by BMI and percentage body fat) at the age of 7 years [27]. The source of protein and timing also matters as a study has shown that dairy protein in particular at 12 months of age is associated with increased adiposity at age 7 years [28]. Furthermore, a study indicated that a positive relation between height and body fatness could be a result of excess energy stored (in the form of fat) which was more than the required amount needed for linear growth [14].

Some other biological factors in infancy and childhood have been shown to promote linear growth. For example, Ong *et al*. have demonstrated that children who displayed catch-up growth in the first two years of life had greater adiposity and were taller for their age compared with those infants who grew at a slower rate and did not display catch-up growth [29]. Also, a recent study has shown that rapid weight gain early in life was associated with higher stature, which itself predicted overweight status at age 3 years [30].

In a review by Scacchi *et al.*, it is indicated that insulin-like growth factor 1 (IGF-1) and growth hormone (GH) reflect nutritional status and that obese children have a higher level of IGF-1 and a decreased level of GH [31]. High protein intake has been suggested to account for this increase in the secretion of IGF-1 [28], and the high level of IGF-1 has the ability to promote adipose tissue hyperplasia and growth [32]. In addition, decrease in GH levels caused by excess protein intake could lead to a reduction in its lipolytic effect [31] hence leading to an increased accumulation of body fat.

The limitations of this study are worth mentioning. This study was carried out exclusively with school-age children in one (North West Region) out of the ten administrative regions of Cameroon. Therefore, the prevalence estimates may not be representative for the whole country. Also, the reference data for waist circumference refer to UK children and it is unclear whether this can be applied to Cameroonian children. In addition, care should be taken in the interpretation of waist circumference findings because studies have shown that an enlarged spleen (which can be asymptomatic) is common in malaria endemic areas [33]. This study did not carry out any malaria diagnosis; therefore, there is the possibility that some WC readings may have been falsified in children who may have an enlarged spleen as a result of malaria. Furthermore, the assessment of pubertal development was not carried out. This could have potentially confounded the association between height and obesity especially at the upper end of age distribution (10 – 12 years) in girls. It is worth noting that because the data in this study was analyzed as a simple random sample, it’s likely that the standard errors were underestimated.

## Conclusions

This study has shown that the children who are taller for their ages tend to have higher adiposity levels; a relationship which is positive and linear not only by using BMI but also WC for both boys and girls. However, a more precise method for determining body fatness like bioelectric impedance analysis (BIA) and a more objective monitoring of height through longitudinal studies may be needed to substantiate these findings.

## Abbreviations

BMI: Body mass index
WC: Waist circumference
SDS: Standard deviation score
BIA: Bioelectric impedance analysis
ANOVA: Analysis of variance
